# A case report: recurrent anemia related to long term acyclovir use in a pregnant HIV infected Ugandan

**DOI:** 10.4314/ahs.v24i1.11

**Published:** 2024-03

**Authors:** Mariam Nambuya, H Mayanja-Kizza

**Affiliations:** Department of Medicine, School of Medicine, Makerere University, P. O. Box 7072 Kampala, Uganda

**Keywords:** Recurrent anemia, acyclovir, herpes genitalis, HIV, ART

## Abstract

This case report describes a pregnant patient with recent diagnosis of Human Immuno-Deficiency Virus (HIV) infection initiated on Anti-Retroviral Therapy (ART) in the second trimester, as well as high dose acyclovir high for large infected genital warts. She had no other HIV related opportunistic infections, and no prior anti tuberculosis treatment or preventive medication. Despite little response to acyclovir, patient was continuing on acyclovir for over 4 months. She subsequently developed recurrent anemia requiring frequent transfusion (14 units in total) over a 6-week period.

On stopping acyclovir, the anemia subsided, a few weeks later she had a normal delivery, followed by surgical removal of the warts. At a follow-up 8 months later, she was well, with a healthy baby, and reported no other episodes of blood transfusion.

## Introduction

Recurrent anemia is not uncommon in pregnancy; and is also associated with HIV infection and ART. In addition, viral infections, and various drugs have been associated with recurrent anemia.

Often teasing out a possible cause in young pregnant woman with HIV infection on ART and with herpes genitalis infection and long-term acyclovir can be complex. In a setting of limited investigative resources, it is even more of an enigma. Often possible cause of recurrent anemia in this scenario may have to be made on exclusion.

Here we report a patient with recurrent anemia which started after initiation of acyclovir, and abated when the drug was discontinued.

It brings to light the importance of careful follow-up of a patient especially where adequate laboratory investigations are not accessible.

## Case

ZN, a 19-year-old female Ugandan presented on 29th October 2019 at the hematology unit of the Mulago-Kiruddu medical unit, an annex of the Mulago national referral hospital in Kampala, Uganda. She had been transferred from the pulmonology unit of the same hospital for management of persistent anemia.

ZN was a prim gravida diagnosed with HIV infection four months previously during an antenatal visit, and commenced on ART (TDF/3TC/EFV). She also had infected bleeding genital warts for which she had been started on oral acyclovir at a dose of 400 mg three times a day at the same time. In addition, she was managed with none specified short term antibiotics by various health care providers.

Five weeks prior to the hematology unit presentation, she was admitted at a community health unit complaining of malaise, weakness, dizziness and headache. She was then 30 weeks pregnant. A diagnosis of malaria and anemia in pregnancy were made, on ART and acyclovir; and she received a quinine therapy, and 5 units of blood over the next 2 weeks.

The anemia however persisted, and was referred to a district level hospital where she was managed as anemia in pregnancy, and received another 4 units of blood.

Due to poor response to treatment, ZN was referred to the national referral women's hospital in Kawempe division of Kampala city. In addition to the HIV and anemia in pregnancy, she was noted to have infected genital warts and a productive cough. She had by then been on acyclovir for 4 months on a continual basis at the same dose as at initiation, which she continued as” self-medication” since the genital warts had not regressed. At the women's hospital, she was advised to continue oral acyclovir as other treatment modalities were not an option in pregnancy. She was transfused with another 3 units of blood, and then referred to Mulago-Kiruddu medical hospital in view of the recurrent severe anemia, and a concerning cough.

On the Kiruddu pulmonology unit, she was noted to have a productive cough for 2 weeks, but investigations for pulmonary tuberculosis (PTB) were negative on sputum smear and Genexpert, as well as having a normal CXR. She was also noted to have recurrent anemia associated with yellowing of the eyes and tea colored urine. By this time, she had received a total of 12 units of blood over a 5 weeks' period. After ruling out tuberculosis, she was transferred to the Kiruddu hospital hematology unit. On further inquiry, she denied history of taking alcohol or cigarette smoking. She was unemployed and had dropped out of school on getting pregnant.

She was still continuing on Acyclovir with a dose of 400mg three times daily up to time of admission to the hematology unit.

On examination ZN was a sick looking young female, about 35 weeks pregnant by fundal height, and with a rather low affect. She had severe pallor, and a tinge of jaundice, mild dehydration but no fever (temperature 370C), and no edema or clinical weight loss.

Her pulse rate was 104 beats/min, blood pressure 90/54 mmHg and respiratory rate of 35 breaths/min.

She had large friable ulcerated genital warts, but with no obvious secondary infection. Review of other systems was unremarkable, in particular the chest was clear. Abdominal examination showed a FH 35/40 and a normal fetal heart, and no enlarged organs. She also had infected soiled ano-genital warts. A diagnosis of severe recurrent anemia, HIV on ART, infected genital warts in pregnancy on 5 months' acyclovir was made.

Hemogram showed hemoglobin (Hb) 4.1 g/dl (normal 8-17), total RBC count 1.54 million cells/mm3 (normal 2.5-5.5) MCV-94.8 fl (normal 80-95) MCH 26.6 (normal 26-38) MCHC 28.1(31-37) Total leucocyte count was 5.07 cells/dl (normal 3-15) platelet count 316/dl. Peripheral blood film showed marked erythrocyte hypochromasia, anisocytosis, macrocytes, few poikilocytes and 5% normoblasts but no inclusion bodies The platelets showed giant forms, and white blood cells were normal. Liver functional tests were not done due to patient financial constraints. She was given a further 2 units of blood (making a total of 14 units over a 6-week period).

A suspicion of acyclovir induced “megaloblastic” anemia was considered at this time. The patient was advised to stop the acyclovir tablets, continued on ART and was given oral and topical antibiotics for the ano-genital warts. She was reluctant to stop acyclovir, and was counselled on the potential effect on the recurrent anemia. She was also counselled on HIV treatment and ART compliance, especially during pregnancy and thereafter.

On review 4 days later, the post transfusion Hb was 7.2 (after the last 2 units of blood) (a total of 14 units over 6 weeks' period by then). Peripheral blood film showed normal white blood cells, anisocytosis, and normal platelets. A complete blood count showed WBC 7.8, RBC 4.26 million cells/mm3 platelets 354/dl, MCV 94.6 fl, MCH 28.6, MCHC 30.9. She was discharged a week after from the hematology ward for review as an outpatient.

At the 2-week follow-up visit, ZN reported to be well with no symptomatology of anemia, and was off acyclovir. She was then referred back to the obstetric unit with advice not to resume acyclovir.

ZN was thereafter followed up in the community. On 04 August 2020, she was traced by mobile telephone review at her mother's home. ZN had had a normal delivery at Kawempe women's hospital, and was currently on regular ART at a district hospital. She never resumed acyclovir, and the genital warts had been surgically removed. She was well, and had not had recurrence of anemia, or any further blood transfusion even at the time of the delivery. At this point she gave a verbal mobile phone consent to write up her case for training of others, without her names being included.

## Discussion

This case report describes a young prim gravida, with HIV infection, commenced on ART, as well as acyclovir for genital warts in pregnancy. She had severe recurrent anemia which regressed on halting the acyclovir. The actual type of anemia was not ascertained in this patient, as no detailed hematological workup, or a bone marrow examination was done.

In the literature, there is limited data on acyclovir associated anemia.

Satadal Barik et al in their study on “Megaloblastic anemia as a drug induced disorder” suggested that acyclovir can be a cause of megaloblastic anemia, but further evidence was necessary to determine the mechanism of this drug induced macrocytosis and megaloblastic anemia.

Amos and Amess in a letter to the editor reported 3 cases of acyclovir related megaloblastic anemia, during treatment for herpes simplex encephalitis. They attributed this to acyclovir inhibition of DNA polymerase.

The Hesdorffer and Longo review article analyzed the drug induced megaloblastic anemias, unfolding biochemical processes and gave importance to the most familiar drugs involved in this disorder; acyclovir is not included in the list of potential drugs. In a correspondence to the above review article, Buti S, Sikokis A include acyclovir as a possible cause of megaloblastic anemia.

According to Hesdoffer and Longo, drugs cause megaloblastic anemia by impairing the cellular availability or use of folic acid or vitamin B12. This may occur because of the interference with the absorption, plasma transport or delivery of folate or vitamin B12 competition for reducing enzymes, end product inhibition of co-factor mediated reactions or physical destruction of the vitamins.

Acyclovir is a purine analogue and antagonist, and may thus inhibit synthesis of purines, thereby interfering with hematopoiesis. Hesdoffer and Longo noted that in purine and pyrimidine synthesis, the methyl group is donated by 5,10 methylene tetrahydrofolate by the enzyme dihydrofolate reductase to create purines and pyrimidines which are both building blocks for DNA and RNA. If there's inhibition of the enzyme dihydrofolate reductase by purine analogue (acyclovir) there will be no purine synthesis and therefore no DNA synthesis leading to Megaloblastic Anemia.

Apak H, et al in a case report on chicken pox induced hemolytic anemia suggested the possible cause of varicella virus as a possible cause of hemolytic anemia.

In this case, it's unlikely that the anemia was related to the herpes genitalis infection although it cannot be ruled out. The warts were operated on beyond 3 weeks of stopping acyclovir with no recurrent anemia during that time.

We were also unable to ascertain the mechanism of the recurrent anemia in this patient. No investigations were done to determine whether it was hemolytic or due to bone marrow suppression, a limitation in this case report. In conclusion, although there is no definite casual effect, increasing case reports allude to the relationship between acyclovir and subsequent anemia. On note some of these are among patients with varicella infections, and there is a possibility that the bone marrow toxicity may be related to the virus or even an effect of both viral infection and acyclovir.

Either way, it's important for clinicians to be aware of the possibility of severe anemia among patients receiving acyclovir, as lack of this may have severe consequences for the patient.
